# NetTCR-2.1: Lessons and guidance on how to develop models for TCR specificity predictions

**DOI:** 10.3389/fimmu.2022.1055151

**Published:** 2022-12-06

**Authors:** Alessandro Montemurro, Leon Eyrich Jessen, Morten Nielsen

**Affiliations:** ^1^ Department of Health Technology, Section for Bioinformatics, Technical University of Denmark, DTU, 2800 Kgs., Lyngby, Denmark; ^2^ Instituto de Investigaciones Biotecnológicas, Universidad Nacional de San Martín, Buenos Aires, Argentina

**Keywords:** T cell, epitope, deep learning, TCR specificity, neural network

## Abstract

T cell receptors (TCR) define the specificity of T cells and are responsible for their interaction with peptide antigen targets presented in complex with major histocompatibility complex (MHC) molecules. Understanding the rules underlying this interaction hence forms the foundation for our understanding of basic adaptive immunology. Over the last decade, efforts have been dedicated to developing assays for high throughput identification of peptide-specific TCRs. Based on such data, several computational methods have been proposed for predicting the TCR-pMHC interaction. The general conclusion from these studies is that the prediction of TCR interactions with MHC-peptide complexes remains highly challenging. Several reasons form the basis for this including scarcity and quality of data, and ill-defined modeling objectives imposed by the high redundancy of the available data. In this work, we propose a framework for dealing with this redundancy, allowing us to address essential questions related to the modeling of TCR specificity including the use of peptide- versus pan-specific models, how to best define negative data, and the performance impact of integrating of CDR1 and 2 loops. Further, we illustrate how and why it is strongly recommended to include simple similarity-based modeling approaches when validating an improved predictive power of machine learning models, and that such validation should include a performance evaluation as a function of “distance” to the training data, to quantify the potential for generalization of the proposed model. The conclusion of the work is that, given current data, TCR specificity is best modeled using peptide-specific approaches, integrating information from all 6 CDR loops, and with negative data constructed from a combination of true and mislabeled negatives. Comparing such machine learning models to similarity-based approaches demonstrated an increased performance gain of the former as the “distance” to the training data was increased; thus demonstrating an improved generalization ability of the machine learning-based approaches. We believe these results demonstrate that the outlined modeling framework and proposed evaluation strategy form a solid basis for investigating the modeling of TCR specificities and that adhering to such a framework will allow for faster progress within the field. The final devolved model, NetTCR-2.1, is available at https://services.healthtech.dtu.dk/service.php?NetTCR-2.1.

## Introduction

T cells form the cornerstone of the adaptive immune system orchestrating and executing attacks on pathogens and pathogen-infected/malfunctioning cells (1, 2). T cell interacts with pathogen or self-aberrant derived peptides (p) presented on the cell surface by MHC (Major Histocompatibility Complex) molecules. This interaction is mediated *via* the trans-membrane T cell receptor (TCR). Not all MHC-presented peptides are able to form an interaction with TCR, and vice versa individual TCRs form a highly specific interaction only with a limited repertoire of pMHC complexes. Understanding the rules underlying this interaction thus holds promise for furthering our understanding of T cell immunogenicity, T cell tolerization, and T cell cross-reactivity.

The TCR is a heterodimeric protein, most often formed by an α- and β-chain. The interaction of TCRs with the cognate pMHC target is primarily defined by 6 loops, 3 on each chain denoted CDR1-3 (complementarity determining regions 1-3). Of these loops, CDR3 interacts primarily with the peptide, and CDR1 and CDR2 primarily with the α loops of the MHC complex (1, 2). The diversity of TCRs is focused mainly on the CDR3s, a region defined by the genomic recombination of the variable, diversity (for CDR3β only), and joining (VDJ) TCR genes.

Large efforts have been dedicated over the years to develop assays for high throughput identification of peptide-specific TCRs. Most of these techniques and assays have focused on sequencing the CDR3β segment, applying cell sorting followed by bulk repertoire sequencing (3, 4). While such approaches are highly cost-effective, they suffer from a relatively high proportion of wrongly identified TCR (present due to carryover in the sorting step). However and more importantly, they suffer from limited information capture and they only describe the CDR3β part of the TCR interaction. We and others have demonstrated the important shortcoming of this limited view on the TCR-pMHC interaction and demonstrated how the information on the specificity of the TCR toward its cognate pMHC target is carried by CDR3 of both α- and β-chains (5, 6). A solution to this is to apply single-cell sequencing enabling the identification of paired α- and β-chains.

A large plethora of methods has been published within the field of prediction of TCR-pMHC interactions. Given this limited amount of paired TCR α- and β data available, the majority of these have focused on CDR3β information only (7–9). Recently however, models are benefitting from the growing volume of paired TCR data allowing for boosting performance by integrating information from both chains (6, 10, 11).

Data on TCR specificity is available in several public databases including VDJdb (12), IEDB (13), McPAS-TCR (14), and TBAdb (15). These databases are highly biased towards data on positive TCR-pMHC interactions. Furthermore, TCR data sets are often highly redundant and composed of many highly similar sequences. Both of these properties pose a challenge when it comes to developing and performance evaluating machine learning (ML) models. In terms of negative data, different approaches have been suggested including mispaired negatives and/or data from healthy controls (7, 16). Most works within TCR specificity have paid very limited attention to data redundancy and sequence similarity, meaning that often the issue has been accessed by only removing identical data points (17, 18). This is clearly an oversimplification, and we have earlier proposed an approach based on the Levenshtein similarities, Hobohm-based redundancy reduction, and single-linkage clustering, and have demonstrated how such careful redundancy considerations can aid the development of models with improved power for generalization (11).

Another critical aspect of TCR-pMHC interaction prediction is the choice between peptide- and pan-specific models. Peptide-specific models are, as the name indicates, models trained specifically for individual peptides, whereas pan-specific models are models encompassing all peptides in the given training data into a single model. Ideally one would seek to develop pan-specific models since these in principle would allow for ab-initio predictions for novel peptides not included in the training data by extrapolation from information and patterns learned across the different peptides. However such extrapolations might only be possible when the coverage of the peptide space in the training data reaches a certain limit. Anecdotally, this is in line with what was observed for the modeling of HLA-peptide binding. Here, HLA-specific models were found to outperform the early pan-specific models and only when the HLA coverage was increased did the pan-specific models perform the best (19). For TCR specificity, modeling the coverage of the peptide space is highly limited, and it hence remains an open question as to whether or not pan-specific models can demonstrate boosted performance.

TCR specificity is as described above defined by the combined signal contained within all 6 CDR loops. Most prediction models have however focused only on the CDR3 loops (and many as stated above only on CDR3β). We have earlier demonstrated how a simple similarity-based model could benefit from the incorporation of information from CDR1 and CDR2 (5), but the overall importance of expanding the CDR information in the context of ML models remains to be settled.

Finally, the development of ML methods within TCR specificity prediction is challenged by the lack of a well-defined baseline model for assessment of ML model performance increase and justify the application of more complex model architectures. Given the very short length of CDRs, usually consisting of 5-25 residues, and the stochastic nature of the generation of in particular CDR3, commonly used evolutionary-based alignment methods cannot be applied here.

Here, we set out to investigate these fundamental questions for the optimal development of TCR specificity prediction models. It is essential to underline that we are not seeking to benchmark different published methods, but that we are solely seeking to address and answer questions related to best practices for developing and evaluating TCR-pMHC models. This with the purpose of aiding the field as a whole, by establishing a foundation and best practice for future work allowing researchers to avoid repeatedly addressing these fundamental issues, and rather focus on developing novel ideas enabling faster progress.

## Materials and methods

### Data preparation

The initial datasets were collected from IEDB, VDJdb, McPAS and 10X Genomics Single Cell Immune Profiling of four donors (20). The original dataset consisted of 21,121 unique paired TCRs relative to 499 peptides and 14 different HLA molecules. Non-binding peptide-TCR pairs were obtained from the 10X dataset. In the 10X assay, T cells were exposed to a panel of 50 peptide-MHC multimers. A negative TCR is defined as a TCR that does not bind any of the tested peptides and that has a Unique Molecular Identifier (UMI) count of 0.

Only data points with both CDR3 α- and β-chains and V/J gene annotations were kept. Further, any cross-reactive TCRs were removed, and the data was restricted to TCRs with CDR3α/β lengths in a range from 6 to 20 amino acids. Finally, only peptides with at least 100 positive TCRs were considered (11). After these initial cleaning steps, the dataset contained 4,111 positive peptide-TCR instances, spanning 10 different peptides and 4 HLA molecules. The negative pool of TCRs counted 40,949 TCRs negative to 6 out of the 10 peptides present in the positive set. The positive TCRs specific to the four non-overlapping peptides were discarded.

The set of positive TCRs was redundancy-reduced with the Hobohm 1 algorithm (21) applied to the CDR3 α- and β-sequences. The TCRs were first sorted in descending order according to the sum of the CDR3α and CDR3β sequence lengths. Briefly, the Hobohm 1 algorithm starts by placing the first TCR into the non-redundant list. Iteratively, all the TCRs are similarity scored against the list of non-redundant TCRs: if the similarity to all the non-redundant TCRs is less than a specified threshold, then the new TCR is assigned to the non-redundant list, otherwise it is discarded. The similarity between sequences was calculated using the kernel similarity measure as defined in (22) and was calculated as the average of the CDR3α- and β-similarity scores. For the positive set, a threshold of 0.95 was chosen to ensure that only highly similar entries were removed. A similar approach was used to reduce the set of negatives, but with a similarity threshold of 0.9. After running the Hobohm 1 algorithm, 3,400 positive and 36,366 negative TCRs were left in the two data sets.

Once the redundancy in the positive set was reduced with the Hobohm 1 algorithm, the data points were randomly split into 6 partitions, 5 for cross-validation and one for external evaluation. For each partition, for each positive peptide-TCR combination, 5 TCRs were sampled from the pool of negative TCRs and added to the partition of the peptide-TCR. These negatives are referred to as true negatives or 10X negatives. Each partition was further augmented with swapped negatives. Here, each positive TCR was paired with 5 peptides (different from the target peptide) and labeled as swapped negative.

The last step in the data curation was to reconstruct the full TCR sequences and annotate gene usage in the CDR loops. First, the full TCR sequences were constructed from V/J genes + CDR3: the CDR3 sequence was merged on the C-terminus of the V gene by looking for a cysteine (C) in the last six residues of the V gene sequence and on the N-terminus by matching a phenylalanine (F) or a tryptophan (W) followed by a glycine (G) within the first 11 amino acids of the J gene sequence. Lastly, Lyra (23) was used to annotate the CDR1 and 2 loops. A total of 473 positive TCR sequences were removed in this step, due to a failure in the TCR reconstruction or CDR annotation.

The final dataset consists of 2,541 unique positive peptide-TCR pairs, 12,848 negatives from 10X and 12,705 swapped negatives. A summary of the peptides included in the training set is shown in **Table 1**.

**Table 1:** Description of the peptides included in the training set.

Peptide Sequence	Organism	HLA	# positive TCRs
GILGFVFTL	Influenza A virus	HLA-A*02:01	969
RAKFKQLL	Epstein Barr virus	HLA-B*08:01	659
ELAGIGILTV	Melanoma	HLA-A*02:01	316
IVTDFSVIK	Epstein Barr virus	HLA-A*11:01	275
GLCTLVAML	Epstein Barr virus	HLA-A*02:01	173
NLVPMVATV	Human CMV	HLA-A*02:01	149

### Baseline model

A baseline model was used to benchmark the performance of the NetTCR model. The baseline used here was inspired by (9) and is solely based on TCR similarities. As for TCRmatch, the kernel similarity (22) measure was used. Briefly, this measure assigns a similarity score between two sequences by comparing all the *k-*mers, with *k* ranging from 1 to the length of the shortest sequence. For a fixed value of *k*, the BLOSUM62 score of all the *k-*mers from the first sequence against the *k-*mers from the second sequence is computed. The similarity score is then given by the self-similarity normalized sum of all the BLOSUM scores, for all the values of *k.*

For each peptide, a database of positive TCRs to the peptide from the training set was constructed and a query with positive and negative (both 10X and swapped negatives) TCRs from the evaluation set. Each TCR in the query is scored against the database using the kernel similarity score. The prediction for a given TCR in the test set is then given by the similarity score to the nearest neighbor in the training set. For the CDR3 model, the similarity score is calculated as the average of the similarities of α- and β-chains. When adding CDR1 and 2 to the model, the overall similarity is calculated as a weighted average of the similarities of each of the 6 CDR loops (3 for the α- and 3 for the β-chain) using weights [1,1,4] and [1,1,4] as suggested earlier (5). It should be noted that the baseline model is inherently peptide-specific as databases and queries are constructed for each peptide separately. TCRbase-1.0, a web server version of the baseline model, is available at https://services.healthtech.dtu.dk/service.php?TCRbase.

### NetTCR model

NetTCR is a sequence-based 1D-convolutional neural network, similar to the one we have earlier proposed in (11). The inputs to the network are the amino acid sequences of the six CDR loops; for the pan-specific model, also the peptide sequence is used as input to the network. The inputs are zero-padded to the left, to ensure the same lengths across input: 10 for CDR 1 and 2, 20 for CDR3, and 13 for the peptides. The sequences are encoded using the BLOSUM50 (24) encoding scheme, mapping each amino acid into a vector with 20 entries. The encoded sequences are processed independently by different convolutional blocks. Each block applies 1D convolutions with 16 filters and kernel sizes {1, 3, 5, 7, 9} (80 filters for each sequence in total). The outputs of the convolutional layers are max-pooled across the sequence length dimension and concatenated. The final part of the network consists of a hidden layer with 32 neurons and an output layer with a single neuron, giving the binding score of the input peptide and TCR. The sigmoid activation function was used in all the layers of the network.

### Model training

All models were trained using nested 5-fold cross-validation for 200 epochs with early stopping, monitoring the validation loss. Adam optimizer was used, with a learning rate of 0.001. The code was developed in Python 3.7; the neural networks were designed using Pytorch 1.11 and the models were trained on an NVIDIA^®^ GeForce GTX TITAN X GPU.

### Performance evaluation

The predictive power of the models was measured using the area under the receiver operating characteristic curve (AUC) and AUC 0.1, defined as the normalized area under the ROC curve with a maximum false positive rate of 0.1. The performance was assessed also with Positive Predictive Value (PPV), defined as the proportion of positive labeled TCRs within the top *n* predictions, where *n* is the number of positive data points in the set.

Each proposed model was trained using nested 5-fold cross-validation resulting in 20 individual networks. The performance was assessed on the left-out evaluation set. Here, the ensemble of the 20 trained models was used and the evaluation predictions were calculated by the average of the predictions from each of the 20 models.

The performance of the models was evaluated in a per-peptide manner (i.e from the subset of TCRs with target values towards a given peptide). For each model, an overall performance was also given by the average AUCs across peptides. We reported the average AUCs both as a mean value of the AUCs from each peptide and as a weighted average of the peptide AUCs, weighted by the number of positive TCRs for that specific peptide in the evaluation set. Each model’s performance was reported by analyzing two tasks: i) positives versus 10X negatives prediction; ii) positives versus swapped negatives prediction.

To overcome the problem of having peptide-specific prediction biases, we performed calibration by transforming the raw prediction scores into percentile rank scores. The rank scores were estimated using a set of 13,847 COVID-specific TCRs (25), not sharing any overlap with the training set. Percentile rank scores for a query TCR was next estimated as the proportion of COVID TCRs that scored higher than the considered TCR, in terms of raw prediction score.

To assess whether the differences in performance were significant, a bootstrap test was performed on the AUC values. Given two prediction vectors from two different models to compare, these were sampled *n* times with replacement, with the same size as the original vectors. Given the null hypothesis that the two models performed equally, a p-value was calculated as the number of times the AUC of the first model, calculated on the resampled vector, was smaller than the one from the second model, normalized by *n.*

## Results

Here, we set out to investigate three essential questions related to the modeling of TCR specificity namely i) the use of peptide- versus pan-specific models, ii) how to best define negative data, and iii) the impact of model-integration of CDR1 and 2 loops. The three questions were addressed by developing and comparing the performance of simple ML models inspired by the earlier NetTCR architecture trained and tested using cross-validation of data extracted from the public domain.

### Baseline model

**Figure 1:**
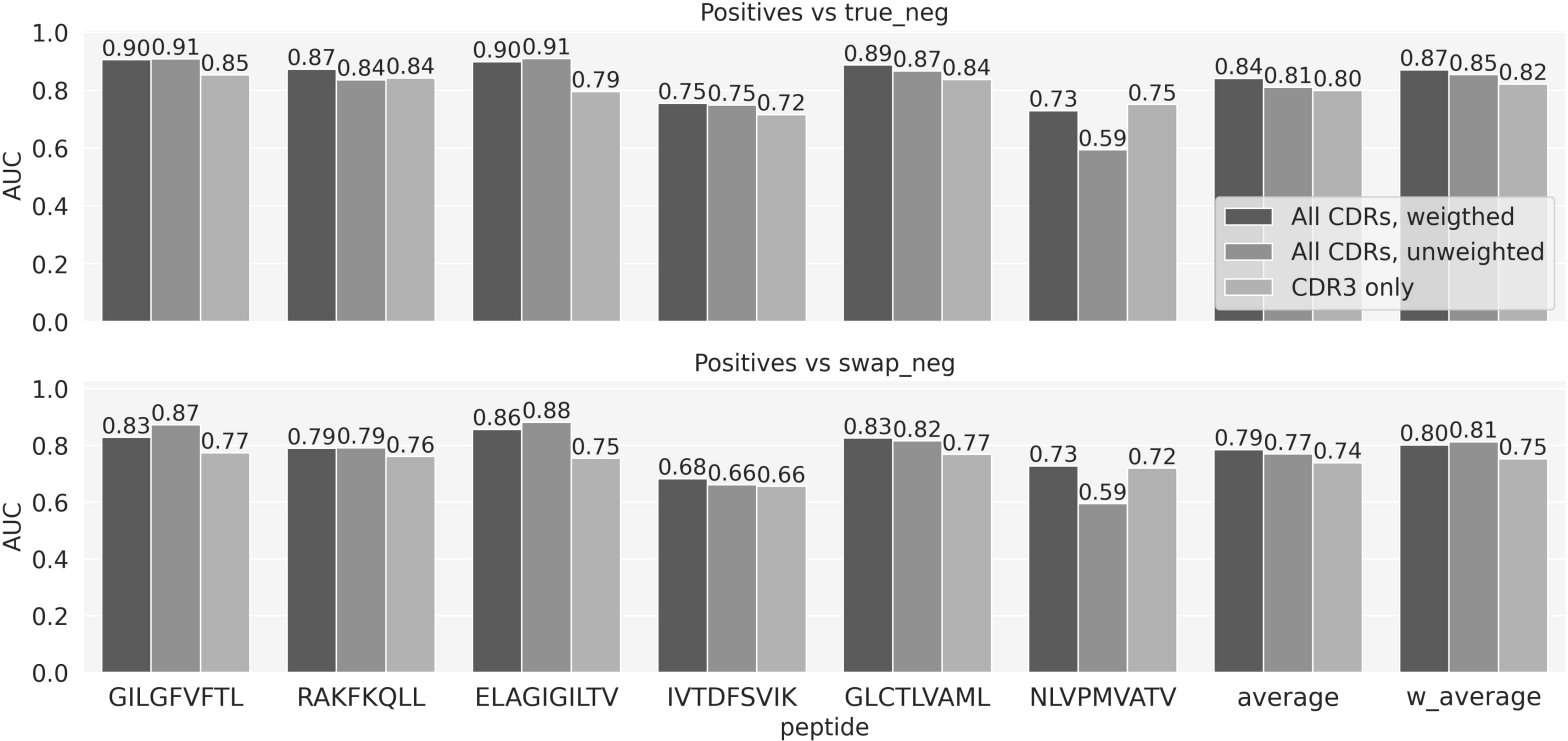
Baseline model performance for weighted and unweighted CDRs. Performance is reported as the AUC for each individual peptide, as well as the average and weighted (by number of positive TCRs) average AUC over the 6 peptides. The performances of three version of the baseline are shown: weighted, where the similarity is given by the weighted sum of the similarities of the three CDRs using the weights [1, 1, 4]; unweighted where all the CDRs are given equal weights; CDR3 only baseline, where CDR1 and 2 are given a weight of 0.

As a baseline model to compare the performance of the more complex ML models, we designed a simple similarity-based model for predicting TCR specificity, TCRbase-1.0, under the assumption that the TCRs that bind the same epitope share a high degree of sequence similarity. Here, for each peptide, a prediction for both positive and negative TCRs from the evaluation set was obtained by comparing these TCRs to all the positive TCRs for that specific peptide in the training set. The similarity score of two TCRs was given by the weighted sum of the similarities of the single CDR loops (see methods). We experimented with different sets of weights for the CDRs, as shown in **Figure 1** and **Supplementary Figure 1**. These results suggest that including CDR1 and CDR2 results in an improved predictive power of the baseline model (p-value<0.001 for all the peptides except IVT and NLV, based on a bootstrap test on the AUC values, with 1000 repetitions). Note that the low performance of the unweighted TCRbase model on NLV is likely due to the higher diversity of the CDR1 and 2 sequences for this particular peptide, compared to the other peptides in the dataset. The 149 positive TCRs cover a set of 43 V genes for the α-chain and 46 for the β-chain. In contrast, these values for the GIL peptide are 60 and 52 for the set of 969 positive TCR. This increased V gene diversity dilutes the information contained in the CDR1 and CDR2 for this data set, hence compromises the predictive power of the TCRbase model when including these in the scoring. Given the overall improved prediction of the model with CDR3s weighted four times higher than CDR1 and 2, we set these weights to be the default configuration of the baseline model.

### Peptide- vs pan-specific model

Next, we wanted to investigate whether peptide or pan-specific models would yield better performance. Ideally, one would like to train pan-specific models pooling all peptide-TCRs in the training data. Thereby, potentially allowing the model to leverage and transfer information between different TCR-pMHC combinations. Such data leverage is however only expected to be beneficial in situations where binding mode information is shared between peptides.

**Figure 2:**
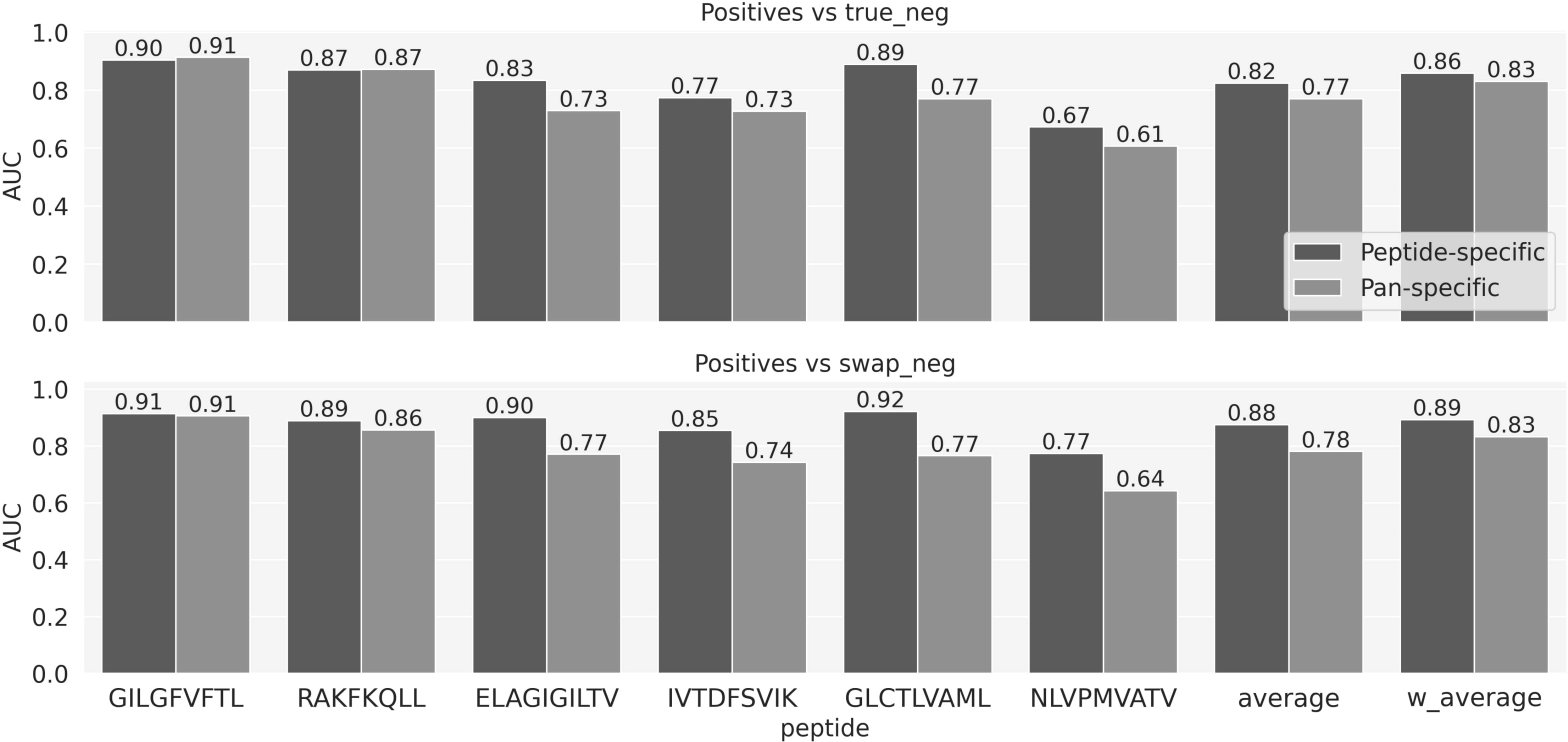
The predictive performance for each peptide measured in terms of AUC of the NetTCR architecture based models trained on α- and β-chains and stratified on negative usage and peptide- versus pan-specific approach. Average and w_aveage denotes the average and weighted (by the number of positive TCRs) average AUC over the 6 peptides.

To compare the predictive power of peptide versus pan-specific models, two sets of models were trained using cross-validation and next evaluated using the left-out evaluation data set (for details see methods). Peptide-specific models were trained for each of the 6 peptides in the training data. The pan-specific model was trained on all data combined. All models were trained using an identical architecture, including the CDR3α and β sequence information from the TCRs, and the peptide sequence as inputs (the peptide information was fully conserved for the peptide-specific models). The result of this experiment is shown in **Figure 2** and demonstrated both for the individual peptides and the combined average performance values that for the data included in this study, the peptide-specific models in the majority of cases achieved superior performance. Particularly for the positives vs swapped negatives prediction task, all the differences are significant, except for the GIL peptide (p-value<=0.01, bootstrap with 1000 repetitions). **Supplementary Figure 2** provides AUC01 and PPV values for the same experiment. Given this, the subsequent work focused only on peptide-specific models.

### On the different sources of negatives

We aimed to investigate the impact of the different sources of negative data points on model performance: 10X negatives and swapped negatives. Briefly, the former set of negatives was derived from the 10X dataset and it is formed by TCRs that were found to not bind any of the 50 tested pMHC multimers. The swapped negatives are artificially generated by pairing TCR sequences with peptides aside from the one to which they were originally annotated to bind.

**Figure 3:**
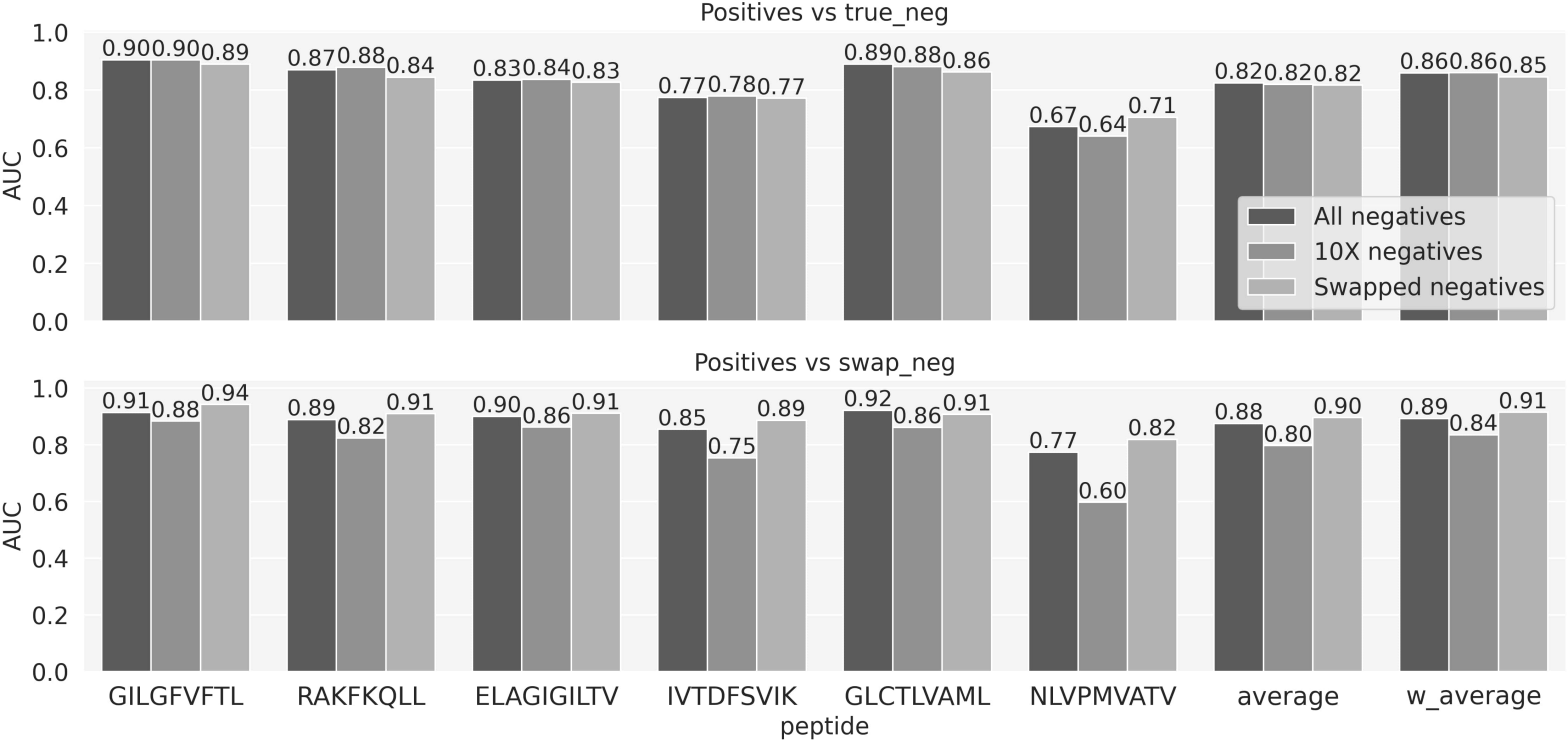
The predictive performance of the three models trained using negatives either from the 10X dataset, the swapped or both combined. The performance is evaluated in terms of AUC on two evaluation sets, each sharing positive observations, but with negatives defined by either true negatives from the 10X dataset or swapped negatives. Average and w_aveage denotes the average and weighted (by number of positive TCRs) average AUC over the 6 peptides.

To investigate the performance impact of the different types of negative data, three models were trained. The first model was trained on the full data, i.e., positives, 10X and swapped negatives. Two more models were trained including either the 10X or swapped negatives. All models were trained using 5 fold cross-validation and evaluated on the 6th independent data set. The results of this experiment are shown in **Figure 3** and **Supplementary Figure 3**, and demonstrated that the models trained on the complete set of negative data overall performed superior compared to the other models. That is, the model trained on the mixed type of negatives outperformed the model trained only on swapped negatives when asked to differentiate between positive and 10X negatives (upper panel). Likewise, it outperformed the model trained on 10X negatives when asked to differentiate between positive and swapped negative (lower panel). Further, the model trained on mixed negatives only suffered a limited decrease in performance when evaluated on the type of negative used to train the two other models. Given these results, we focused on the model trained using mixed negative data moving forward.

### Adding CDR1 and CDR2

**Figure 4:**
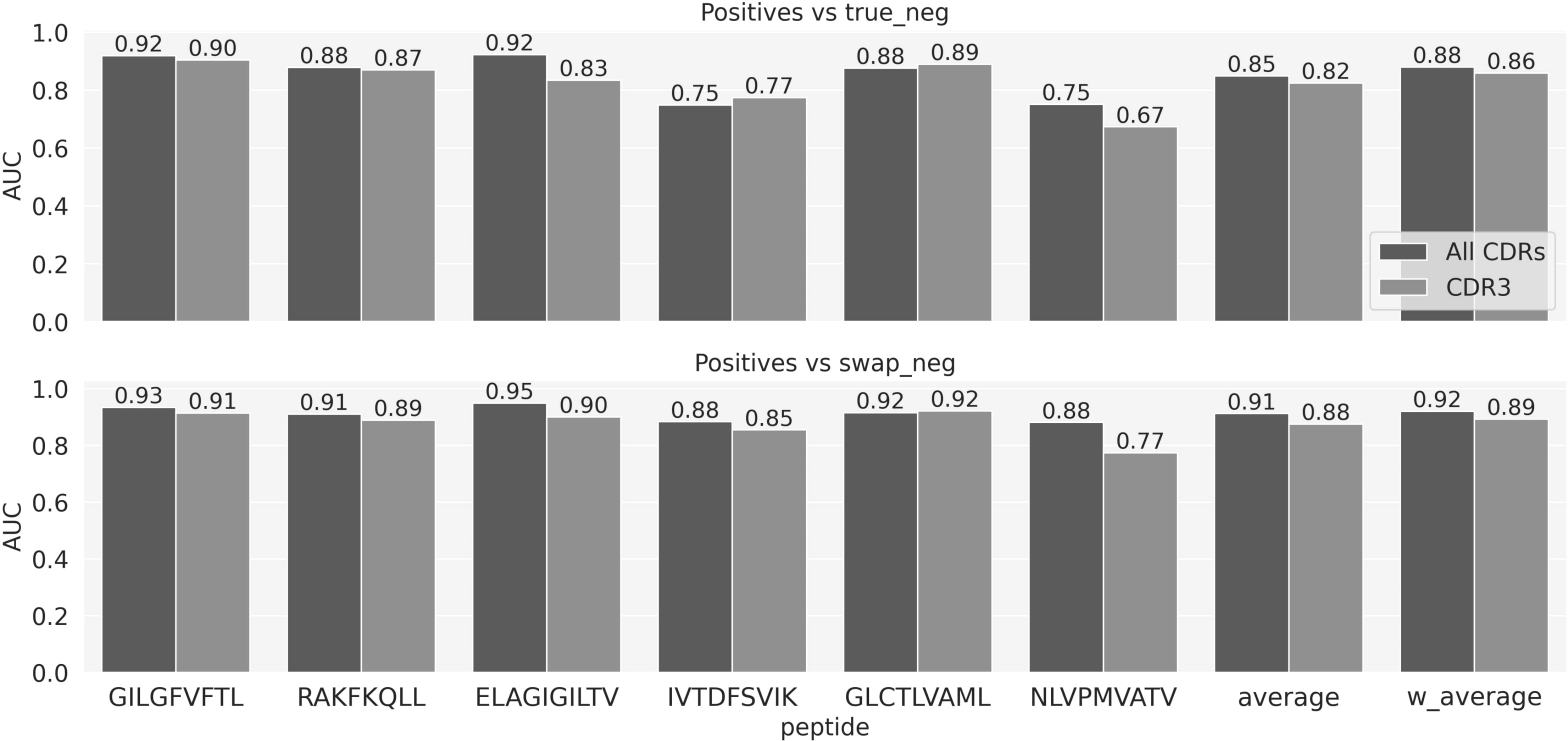
Performance comparison in terms of AUC for the NetTCR model using all CDR loops versus using only CDR3 loops from both α- and β chains.

We next expanded the NetTCR architecture to also include CDR1 and -2 sequences as input, hereby representing the TCR as 6 sequences, the three CDRs from the α chain and the three from the β. **Figure 4** shows the AUCs on the evaluation set of the model with all the CDR and the model with only CDR3s. **Figure 4** demonstrates an overall improved performance when adding the CDR1 and 2. This gain is larger when looking at the AUC calculated on the positive vs swapped negative prediction task (**Figure 4**, lower panel) compared to positives versus true negatives (**Figure 4**, upper panel). Except for the GLC peptides, the model trained on all the CDRs significantly outperforms the one trained on CDR3 only (p-value<0.001, based on a bootstrap test with 1000 resampling with replacement) across all the peptides, when looking at the positives versus swapped negatives prediction. AUC01 and PPV comparisons are shown in **Supplementary Figure 4**.

**Figure 5:**
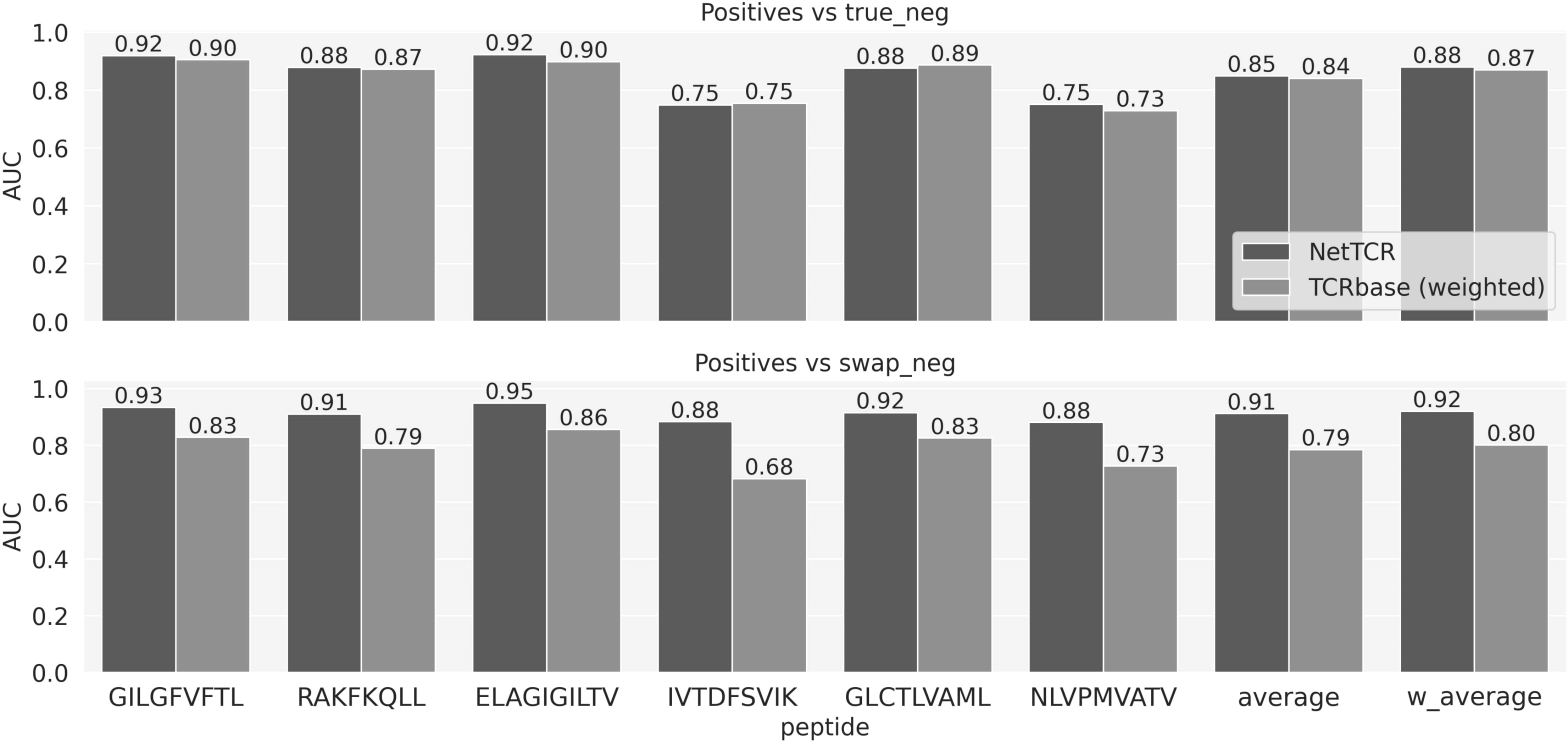
Predictive performance measured in terms of AUC for the peptide-specific NetTCR CDR123 model and the baseline.

Lastly, we compared NetTCR to the baseline model (TCRbase) with weighted CDRs contributions, as shown in **Figure 5** and **Supplementary Figure 5**. The two models achieved comparable performance with a minor advantage of NetTCR when tested on the task of predicting the positive vs 10X negatives (**Figure 5**, upper panel). However, NetTCR significantly outperformed the baseline (p-value <0.001, bootstrap test with 1000 repetitions) for all evaluations when separating between positives and swapped negatives (**Figure 5**, bottom panel).

### Predicting peptide targets

**Figure 6:**
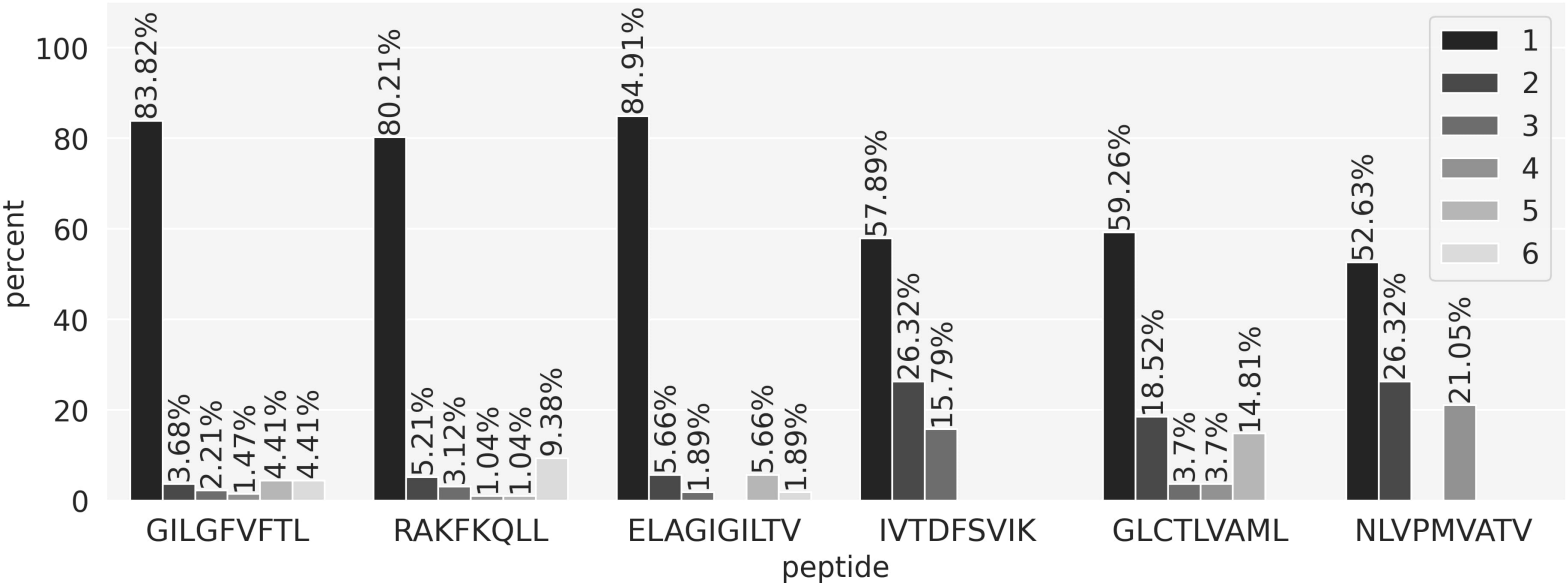
Peptide ranking analysis. Each positive TCR in the evaluation set was paired with all 6 peptides and predictions were obtained using the peptide-specific models. For each TCR, the six prediction scores were sorted in descending order and a rank was obtained. A rank of 1 means that the model correctly predicted the true TCR-peptide pair, assigning the highest score. The bars in the plot show the proportion of TCRs for each rank value.

So far, the performance evaluations performed have focused on evaluating to what degree models can differentiate between TCRs being positive or negative towards a given peptide. Equally interesting is whether a model is capable of identifying the true target peptide from a pool of possible peptides. To evaluate this, we compiled a data set where all the positive TCRs were paired to all the six peptides in the training set. Next, we used the peptide-specific models to get predictions for these peptide-TCR combinations and the scores were sorted in descending order. Ideally, the TCR paired to its target peptide should get a rank of 1, meaning that the prediction score for this true positive combination was the highest among all possible combinations resulting in 0 false positive predictions. The results of this experiment are shown in **Figure 6**. Here, the rank distribution for the positive TCRs for each peptide is shown. Most of the TCRs are observed to get a rank of 1, meaning that they were assigned to the correct peptide and thus received the highest score by the model corresponding to the correct target peptide. In all cases, the rank distributions are improved compared to the uniform distribution of a random model. However, the proportion of top-ranked predictions varied between the different peptides with values above 80% for the three most covered peptides and a drop to around 55% for the three least covered. The number of top 1 positive TCRs for each peptide are GIL 114/136, RAK 77/96, ELA 45/53, IVT 22/38, GLC 16/27, NLV 10/19.

**Figure 7:**
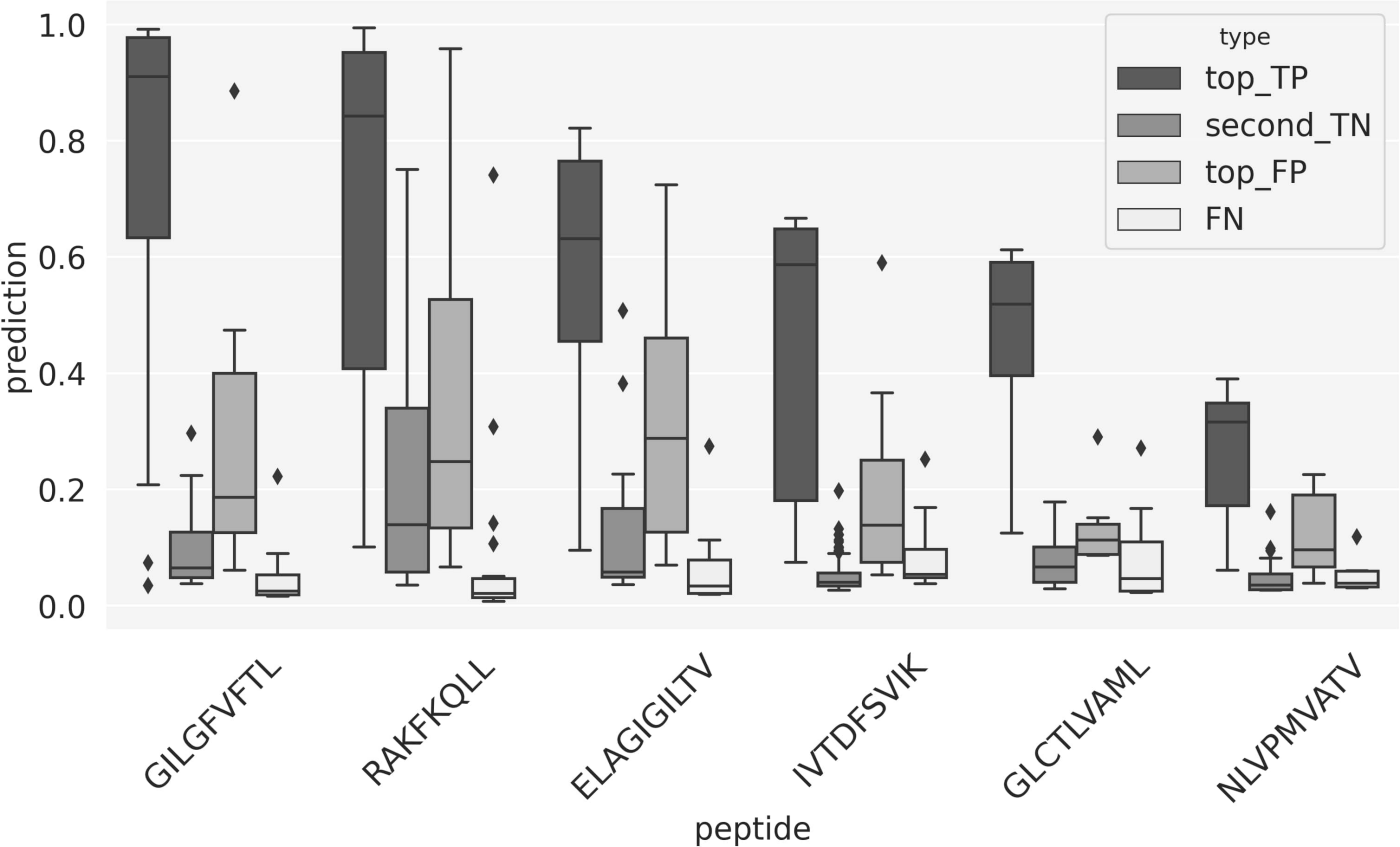
Box-plots of the prediction scores from the peptide ranking analysis. “top_TP” refers to the predictions for the positive peptide-TCRs that obtained the highest prediction score with the model trained on that specific peptide; “second_TN” shows the predictions for the second highest scoring TCR. “top_FP” and “FN” refer to a scenario where a TCRs gets the highest prediction score when paired to a peptide that is different from its target. “top_false_FN” shows the score distribution of these wrong combinations of peptide and TCR; “FN” represents the prediction score of the correct peptide-TCR pairs that did not score top 1.

To further investigate the source of these performance variations, **Figure 7** shows box-plots of the prediction scores for different subsets of TCRs. Here the “top_TP” and “second_TN” refer to scores of the top and second scoring peptide for a given TCR, in the situation where the true peptide is ranked top-one. The other two distributions refer to the case where the model was not able to top-rank the correct peptide for the TCR. Here “top_FP” displays the distribution of the prediction scores for the wrongly predicted top-one peptides, and “FN” is the score distribution for the correct peptide. Comparing the first two box-plots thus informs about the gap in the scores between top one and two in the situation of a correct prediction, and the last two plots about both the overall score distribution for TCRs with wrong predictions and the score of the best peptide in these situations. Several important conclusions can be drawn from these plots. First and foremost are the score distributions for “top_TP” and “second_TN” in all cases very well separated, suggesting that in these cases, the model has high certainty in predicting the correct peptide target. Secondly, variations in score distribution for the “top_TP” between the different peptides - the median score values decrease as one moves from the highest covered (GIL) towards the least covered (NLV) peptides, suggest that a score calibration would potentially benefit the peptide ranking evaluation. Lastly, the scores for the FN TCR are in all cases very low and distinctively different from the “top_TP” score distributions. This strongly suggests that these FN TCRs at least in part are TCRs, which have been incorrectly annotated. We can pursue this further by investigating the source of the TCRs in the two classes “top_TP” and “FN”. Doing this, we find that one publication (26) in particular is enriched in “FN” TCR. This publication contributes ~19% of the TCRs in the FN category while only contributing ~10% to the overall positive data set and ~5% to the top_TP category. The underlying source of this FN enrichment is unclear.

**Figure 8:**
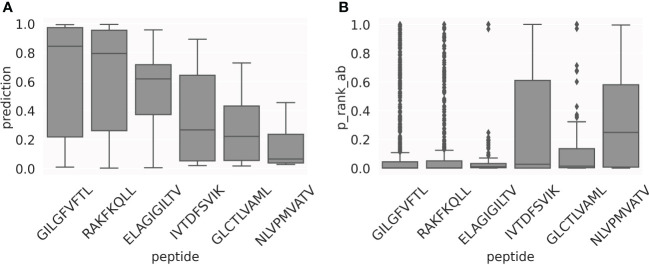
Motivation for using percentile ranks. Box-plots of the prediction scores **(A)** and percentile rank values **(B)** for the set of positive TCRs in the test CV sets.

As illustrated in **Figure 7**, the prediction scores for the top1 TCRs have very different median values, depending on the peptide. In general, this happens for all the positive TCRs, as shown in **Figure 8A**. This represents a limitation when comparing predictions from different models, thereby indicating that a score calibration is needed. To address this, we applied a percentile rank transformation to the raw prediction scores to avoid these peptide-specific scoring biases, as described in Materials and Methods. Here, a set of 13,847 COVID-specific TCRs (25) were used to estimate the background distributions of the peptide-specific models. The percentile rank score for a peptide-TCR pair in the evaluation set was then estimated as the proportion of the background COVID TCRs with a higher prediction score than the pair in consideration. **Figure 8B** shows the percentile rank scores for the positive TCRs. Except for the NLV peptide, the median values of the percentile rank scores are now comparable across peptides. This suggests that using the percentile rank scores is more appropriate than using the raw prediction scores, making the different models more directly comparable.

### Performance as a function of distance to training data

**Figure 9:**
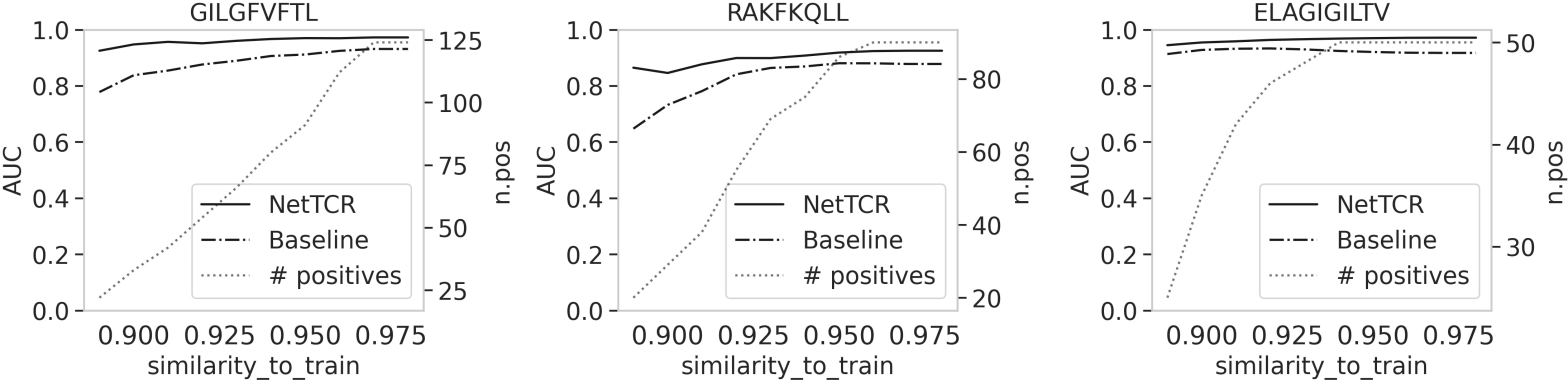
AUC values as a function of the similarity between training and evaluation set. Percentile rank transformation was applied to the TCRs and only positive TCRs with a rank score less than 0.3 were kept in this analysis. For each TCR in the evaluation set, we calculated the similarity to the training set using the kernel similarity score. We then removed the TCRs with a similarity above a threshold and calculated the AUC. The curves in the plots show the AUC varies when different similarity thresholds were used to filter the evaluation set; 10 similarity values between 0.89 and 0.98 were chosen. The dashed line shows the number of positive TCRs left in the evaluation set at each step of filtering by similarity to the training set.

Next, we wanted to investigate how the similarity between the training and evaluation set drove the performance of both NetTCR and the baseline models. In these experiments, we exclude positive TCRs with a percentile rank score above 0.3 (to exclude potential noise imposed by the FN TCRs described above). For each TCR, we defined its similarity to the training set as the kernel similarity score to its nearest neighbor TCR, either positive or negative, in the training set. Next, we excluded TCRs with a similarity to train above a given threshold and calculated the AUC value based on the predictions of the remaining data points. **Figure 9** shows the results of this experiment, using different similarity threshold values between 0.89 and 0.98 (results shown for the three most frequent peptides). These results show that when the TCRs in the evaluation set are allowed to share a similarity to the training set up to 0.98, the baseline and NetTCR models perform similarly. However as the maximum similarity between the train and evaluation set is reduced, the gap in performance between the two models increases (in particular for the GIL and RAK peptides), with a substantial drop in baseline AUC for the baseline model, while NetTCR to a high degree maintains performance.

### The NetTCR-2.1 method

The presented model is available as a web-server implementation at https://services.healthtech.dtu.dk/service.php?NetTCR-2.1. The server allows users to make TCR-binding predictions to one or more peptides, using the peptide-specific models. It is possible to use either the models trained on CDR3α- and β-sequences or trained using all the six CDR loops.

The output of NetTCR-2.1 is a list of CDR-peptide pairs along with the binding prediction. For each prediction, the method outputs also the percentile rank score, estimated from a background set of 13,847 COVID-specific TCRs. The percentile rank is a normalized score across the different peptide-specific models, ranging from 0 to 1, where 0 is the best possible percentile rank. The rank score should serve as a guideline to select a peptide invariant threshold on the binding probability prediction. For each peptide, the threshold could be defined as the 75th percentile of the background prediction score distributions (boxplots shown in **Figure 8B**).

## Discussion

Here, we present NetTCR-2.1, which is an extension of our earlier NetTCR-2.0 method for prediction of pMHC-TCR interactions. The main augmentation is an extended peptide coverage and the ability to include all CDRs in the binding prediction.

In our work, we investigated several important aspects of model development when aiming at predicting TCR specificity and have presented our results of this, aiming at supplying the TCR-specificity prediction field with a set of suggested best practices. These include recommendations on strategies for data partitioning and redundancy reduction, the use of peptide versus pan-specific modeling, the source of negatives, inclusion of CDR1 and CDR2 information, the importance of benchmark comparison of simple sequence similarity-based baseline models, and model performance comparison in the context of distance to training data. In the following, we will briefly summarize our findings and associated conclusions on each of these topics.

### Strategies for data partitioning and redundancy reduction

Traditionally, TCR-pMHC specificity data has been focused on CDR3β for reasons previously described. However, the advent of high throughput single cell technologies has resulted in a substantial increase in publicly available data on paired TCR α- and β-chain to cognate pMHC-target data. However, it is evident that these data reflect ongoing research into model organisms or diseases like Influenza A virus, Epstein Barr virus, Melanoma and Human CMV. Furthermore, often only positive observations of TCR-pMHC interaction are reported, biasing the databases. As a reflection of this, the TCR sequences currently available share a high degree of redundancy. Therefore, in order to achieve, to the degree possible, non-biased training and evaluation of the developed models, it is important to address redundancy. This is true particularly for modern modeling frameworks, where parameter space is very large and they are prone to overfitting.

Due to the genetic mechanisms underlying TCR generation, classical alignment-based similarity approaches using for instance Blosum matrices and affine gap penalties are nonsensical. Therefore, we propose using alignment-free methods such as the kernel method described by Shen et al. (22) to estimate sequence similarity and then subsequently perform redundancy reduction using e.g. the Hobohm1 algorithm. Lastly, we recommend performing pre-clustering prior to partitioning the data using e.g. single linkage to ensure the least possible overlap between partitions.

### Peptide versus pan-specific modeling

We trained two versions of the NetTCR model; a pan- and a peptide-specific, both trained only including the CDR3 for simplicity. Ideally, a pan-specific approach should be more generalizable and rely less on the individual peptides in the training set, aiming at capturing the global signal. The clear advantage is that such a model would be able to make predictions for TCRs specific to any peptide, even for those peptides that are represented by only a small sample in the training data or even absent. Given the data currently available, the peptide-specific models were however found to outperform the pan-specific ones. Using the experiences gained from modeling pMHC-interaction where the early pan-specific model also performed at par or slightly worse than allele-specific (27), this is likely due to the limited volume and coverage of the data volume currently available. We observe that TCRs specific to different peptides do not share many features, rendering cross-learning across peptides not achievable at the moment. As more data becomes available, we expect that it will be possible to train pan-specific models.

### The source of negatives

A critical point when developing an ML model for binary classification is the definition of negative data. Insufficient consideration of this can lead to biases in the obtained conclusions (28). The publicly available datasets of TCR-pMHC sequences almost exclusively contain examples of positive binding pairs. Only the recently published 10X Genomics dataset contains both positive and negative data points. Another common approach for generating artificial negatives is to mispair positive peptide-TCR pairs. Here, we have compiled a training data set with both 10X negatives and internal mispairing of peptides and TCRs, referred to as swapped negatives. We investigated the impact of both sources of negatives by training the same neural network models on different datasets, including either both sources or negatives or only one of the two. In all these experiments, the NetTCR CDR3 peptide-specific model was adopted. To better understand how the two negative sets affected the performance, the AUC values were calculated for the positive vs swapped negatives and for the positives vs 10X negatives prediction tasks. The model trained with only swapped negatives showed a high predictive power when evaluating the positives vs swapped negatives, as that was specifically the task the model was trained for. However, evaluating how this model could distinguish between positives and 10X negatives, the performance was observed to drop. Vice versa, the model trained on 10X negatives was demonstrated to obtain high performance for the positives vs 10X negatives task but suffered a major drop in performance when making predictions on the swapped negatives. In contrast, the model trained on the entire dataset, i.e. positive TCRs, 10X and swapped negatives, showed high performance on both tasks of predicting 10X and swapped negatives. These results suggest that both types of negatives contribute to the model performance. Furthermore, given the large drop in performance on the swapped data of the network trained on the 10X negatives the swapped negatives play an essential role in learning how to differentiate between positive and negative TCRs for a given peptide. This aspect could suggest that the positive and 10X negative TCRs form two disjoint sets. Hence, the network might capture a signal to distinguish positives and negatives that are different in sequences, but not learn the rules that make a TCR positive to one peptide and negative towards others.

### Inclusion of CDR1 and CDR2 information

Most of the available models to predict peptide-TCR interaction are focused on CDR3β or paired CDR3αβ sequences, and only a few recently published works have added V/J genes information in the model as one-hot encoded features (6, 10). Here, we have developed a neural network that takes as input the full set of the 6 CDR sequences. The full-length TCR was reconstructed from the V/J genes and CDR3 sequence, and the CDRs were annotated using Lyra (see Material and Methods for details). We compared the model trained on the full set of CDRs to the one trained on CDR3αβ data. On average, the model trained on the 6 CDR sequences showed higher AUC values compared to the CDR3αβ model, across the peptide set. For some of the peptides, the inclusion of CDR1 and 2 resulted in a substantial increase in AUC. This is the case, for instance, for the ELAGIGILTV peptide. However, this gain in performance might be driven by a bias in the V gene data. In our data set, 85% of the positive ELA CDR1α and 2α are encoded by the TRAV12-2*01 gene; this gene is present only in a minor proportion (5%) in the negative set. It is not clear if this bias is due to the data collection or if it is a biological signal.

### Benchmark comparison of simple sequence similarity-based baseline models and models comparison in the context of distance to training data

Together with NetTCR-2.1, we have here proposed TCRbase, a similarity-based approach to predict TCR-peptide interaction, under the assumption that TCRs with similar sequences recognize the same epitope. We showed that this model achieved comparable performance to the one of NetTCR, while being very simple. These results align with previous findings (17, 26, 29, 30). A closer analysis of our results revealed that TCRbase performed at par with NetTCR when separating positive versus 10X negative TCRs; however, the gap in performance between the two models was enlarged on the positives versus swapped negatives prediction task, where NetTCR significantly outperformed TCRbase. This behavior suggests that the 10X negatives are very different from the positive TCRs, and this dissimilarity makes it trivial for a similarity-based model to distinguish between positives and negatives. This is not the case for the swapped negatives, as they are positive to some other peptide. Here, TCRbase to a higher degree fails in separating the positive and negative set, while NetTCR maintains performance, indicating that the neural network has learned some features beyond sequence similarity. The generalizability of NetTCR is furtherly confirmed when comparing the model’s performances in the context of distance to training data. When the evaluation set is allowed to be highly similar to the training data, NetTCR and TCRbase have comparable performance in terms of AUC. As the TCRs similar to the training data are removed, TCRbase suffers a drop in performance for two out of the three peptides analyzed, whereas NetTCR is able to maintain the predictive power. We believe both of these results are essential as a validation of the greater potential for generalization of the NetTCR machine learning-based method over the more simple similarity-based approach, and strongly suggest that such similarity-based models and performance evaluations as a function of distance to training data are included as baselines in future works developing TCR specificity prediction models.

## Data availability statement

The original contributions presented in the study are included in the article/**Supplementary Material**. Further inquiries can be directed to the corresponding author.

## Author contributions

AM and MN designed the study. The experimental data used in the study was collected by AM. AM generated the computational results and figures, with contributions from LJ and MN. All authors contributed to the methodology and provided scientific feedback. All authors contributed to the article and approved the submitted version.

## Supplementary material

Supplementary Figure 1 Baseline model performance comparison in terms of AUC01 and PPV. The baseline model was used i) with weights [1, 1, 4] on the CDRs; ii) with equal weights on the CDrs; iii) using only CDR3s. The values are given for each peptide, and on the positives vs 10X negatives and positives vs swapped negatives prediction tasks. average and w_average refer to the average and weighted average of the AUC01 (and PPV) across the six peptides.

Supplementary Figure 2 AUC01 and PPV values comparison of the NetTCR model trained in a peptide-specific or a pan-specific manner. Performance reported for each peptide, and for positives vs. 10X negatives and positives vs swapped negatives task. Average and weighted average (weighted by the number of positive TCRs for each peptide) performances are also reported.

Supplementary Figure 3 Analysis of the different sources of negatives. AUC01 and PPV values for the NetTCR-CDR3 model trained on i) the full dataset, including positives, 10x negative and swapped negatives; ii) positives and 10x negatives only, iii) positives and swapped negatives only. AUC01 and PPV are reported in a peptide-specific manner; the values are also differentiated based on positives versus 10X/swapped negatives predictions. “average” refers to the mean values of AUC01 (and PPV) from each peptide; “w_average” is a weighted average (weighted by the number of positive TCRs) of the values.

Supplementary Figure 4 Peptide-specific AUC01 and PPV values comparison of the NetTCR models trained using the set of all CDRs or CDR3 only. The predictive power is evaluated for each peptide (average and w_average refer to an average and weighted average, respectively, of the peptide-specific scores). The performance is also differentiated based on the positives vs. 10X/swap negatives predictions.

Supplementary Figure 5 NetTCR versus TCRbase. Performance comparison in terms of AUC01 and PPV. The values are reported for each peptide, and differentiated according to the two prediction tasks, positives vs 10x negatives and positives vs swapped negatives. “average” is calculated as an average of the AUC01 (and PPV) of the peptide-specific scores; “w_average” is a weighted average (weighted by the number of positive TCRs) of the peptide-specific scores.

## References

[1] Krogsgaard M Davis MM . How T cells “see” antigen. Nat Immunol (2005) 6:239–45. doi: 10.1038/ni1173 15716973

[2] Davis MM Bjorkman PJ . T-Cell antigen receptor genes and T-cell recognition. Nature (1988) 334:395–402. doi: 10.1038/334395a0 3043226

[3] Klinger M Pepin F Wilkins J Asbury T Wittkop T Zheng J . Multiplex identification of antigen-specific T cell receptors using a combination of immune assays and immune receptor sequencing. PloS One (2015) 10:e0141561. doi: 10.1371/journal.pone.0141561 26509579 PMC4624875

[4] Rius C Attaf M Tungatt K Bianchi V Legut M Bovay A . Peptide-MHC class I tetramers can fail to detect relevant functional T cell clonotypes and underestimate antigen-reactive T cell populations. J Immunol (2018) 200:2263–79. doi: 10.4049/jimmunol.1700242 PMC5857646 29483360

[5] Lanzarotti E Marcatili P Nielsen M . T-Cell receptor cognate target prediction based on paired α and β chain sequence and structural CDR loop similarities. Front Immunol (2019) 10:2080. doi: 10.3389/fimmu.2019.02080 31555288 PMC6724566

[6] Zhang W Hawkins PG He J Gupta NT Liu J Choonoo G . A framework for highly multiplexed dextramer mapping and prediction of T cell receptor sequences to antigen specificity. Sci Adv (2021) 7. doi: 10.1126/sciadv.abf5835 PMC8121425 33990328

[7] Springer I Besser H Tickotsky-Moskovitz N Dvorkin S Louzoun Y . Prediction of specific TCR-peptide binding from Large dictionaries of TCR-peptide pairs. Front Immunol (2020) 11:1803. doi: 10.3389/fimmu.2020.01803 32983088 PMC7477042

[8] Gielis S Moris P Bittremieux W De Neuter N Ogunjimi B Laukens K . Detection of enriched T cell epitope specificity in full T cell receptor sequence repertoires. Front Immunol (2019) 10:2820. doi: 10.3389/fimmu.2019.02820 31849987 PMC6896208

[9] Chronister WD Crinklaw A Mahajan S Vita R Koşaloğlu-Yalçın Z Yan Z . TCRMatch: Predicting T-cell receptor specificity based on sequence similarity to previously characterized receptors. Frontiers in Immunology (2020) 12:640725. doi: 10.1101/2020.12.11.418426 PMC7991084 33777034

[10] Springer I Tickotsky N Louzoun Y . Contribution of T cell receptor alpha and beta CDR3, MHC typing, V and J genes to peptide binding prediction. Front Immunol (2021) 12:664514. doi: 10.3389/fimmu.2021.664514 33981311 PMC8107833

[11] Montemurro A Schuster V Povlsen HR Bentzen AK Jurtz V Chronister WD . NetTCR-2.0 enables accurate prediction of TCR-peptide binding by using paired TCRα and β sequence data. Commun Biol (2021) 4:1060. doi: 10.1038/s42003-021-02610-3 34508155 PMC8433451

[12] Bagaev DV Vroomans RMA Samir J Stervbo U Rius C Dolton G . VDJdb in 2019: Database extension, new analysis infrastructure and a T-cell receptor motif compendium. Nucleic Acids Res (2020) 48:D1057–62. doi: 10.1093/nar/gkz874 PMC6943061 31588507

[13] Vita R Mahajan S Overton JA Dhanda SK Martini S Cantrell JR . The immune epitope database (IEDB): 2018 update. Nucleic Acids Res (2019) 47:D339–43. doi: 10.1093/nar/gky1006 PMC6324067 30357391

[14] Tickotsky N Sagiv T Prilusky J Shifrut E Friedman N . McPAS-TCR: A manually curated catalogue of pathology-associated T cell receptor sequences. Bioinformatics (2017) 33:2924–9. doi: 10.1093/bioinformatics/btx286 28481982

[15] Zhang W Wang L Liu K Wei X Yang K Du W . PIRD: Pan immune repertoire database. Bioinformatics (2020) 36:897–903. doi: 10.1093/bioinformatics/btz614 31373607

[16] Jurtz VI Jessen LE Bentzen AK Jespersen MC Mahajan S Vita R . NetTCR: Sequence-based prediction of TCR binding to peptide-MHC complexes using convolutional neural networks. BioRxiv (2018). doi: 10.1101/433706

[17] Chronister WD Crinklaw A Mahajan S Vita R Koşaloğlu-Yalçın Z Yan Z . TCRMatch: Predicting T-cell receptor specificity based on sequence similarity to previously characterized receptors. Front Immunol (2021) 12:640725. doi: 10.3389/fimmu.2021.640725 33777034 PMC7991084

[18] Sidhom J-W Larman HB Pardoll DM Baras AS . DeepTCR is a deep learning framework for revealing sequence concepts within T-cell repertoires. Nat Commun (2021) 12:1605. doi: 10.1038/s41467-021-21879-w 33707415 PMC7952906

[19] Hoof I Peters B Sidney J Pedersen LE Sette A Lund O . NetMHCpan, a method for MHC class I binding prediction beyond humans. Immunogenetics (2009) 61:1–13. doi: 10.1007/s00251-008-0341-z 19002680 PMC3319061

[20] A new way of exploring immunity - linking highly multiplexed antigen recognition to immune repertoire and phenotype | technology networks a new way of exploring immunity - linking highly multiplexed antigen recognition to immune repertoire and phenotype. Available at: https://www.technologynetworks.com/immunology/application-notes/a-new-way-of-exploring-immunity-linking-highly-multiplexed-antigen-recognition-to-immune-repertoire-332554 (Accessed January 20, 2021). 10X Genomics.

[21] Hobohm U Scharf M Schneider R Sander C . Selection of representative protein data sets. Protein Sci (1992) 1:409–17. doi: 10.1002/pro.5560010313 PMC2142204 1304348

[22] Shen WJ Wong HS Xiao QW Guo X Smale S . Towards a mathematical foundation of immunology and amino acid chains. arXiv preprint (2012) arXiv:1205.6031.

[23] Klausen MS Anderson MV Jespersen MC Nielsen M Marcatili P . LYRA, a webserver for lymphocyte receptor structural modeling. Nucleic Acids Res (2015) 43:W349–55. doi: 10.1093/nar/gkv535 PMC4489227 26007650

[24] Henikoff S Henikoff JG . Amino acid substitution matrices from protein blocks. Proc Natl Acad Sci USA (1992) 89:10915–9. doi: 10.1073/pnas.89.22.10915 PMC50453 1438297

[25] Minervina AA Pogorelyy MV Kirk AM Crawford JC Allen EK Chou C-H . SARS-CoV-2 antigen exposure history shapes phenotypes and specificity of memory CD8+ T cells. Nat Immunol (2022) 23:781–90. doi: 10.1038/s41590-022-01184-4 PMC9106845 35383307

[26] Dash P Fiore-Gartland AJ Hertz T Wang GC Sharma S Souquette A . Quantifiable predictive features define epitope-specific T cell receptor repertoires. Nature (2017) 547:89–93. doi: 10.1038/nature22383 28636592 PMC5616171

[27] Nielsen M Lundegaard C Blicher T Lamberth K Harndahl M Justesen S . NetMHCpan, a method for quantitative predictions of peptide binding to any HLA-a and -b locus protein of known sequence. PloS One (2007) 2:e796. doi: 10.1371/journal.pone.0000796 17726526 PMC1949492

[28] Sidorczuk K Gagat P Pietluch F Kała J Rafacz D Bąkała L . Benchmarks in antimicrobial peptide prediction are biased due to the selection of negative data. Brief Bioinf (2022). doi: 10.1093/bib/bbac343 PMC9487607 35988923

[29] Wong EB Gold MC Meermeier EW Xulu BZ Khuzwayo S Sullivan ZA . TRAV1-2+ CD8+ T-cells including oligoconal expansions of MAIT cells are enriched in the airways in human tuberculosis. Commun Biol (2019) 2:203. doi: 10.1038/s42003-019-0442-2 31231693 PMC6549148

[30] Meysman P Barton J Bravi B Cohen-Lavi L Karnaukhov V Lilleskov E . Benchmarking solutions to the T-cell receptor epitope prediction problem: IMMREP22 workshop report. BioRxiv (2022). doi: 10.1101/2022.10.27.514020

