# Supplementary figures and images for: NetTCR-2.1: Lessons and guidance on how to develop models for TCR specificity predictions
Source: Front Immunol. 2022 Dec 6;13:1055151. doi: 10.3389/fimmu.2022.1055151 (PMC9763291; doi:10.3389/fimmu.2022.1055151)

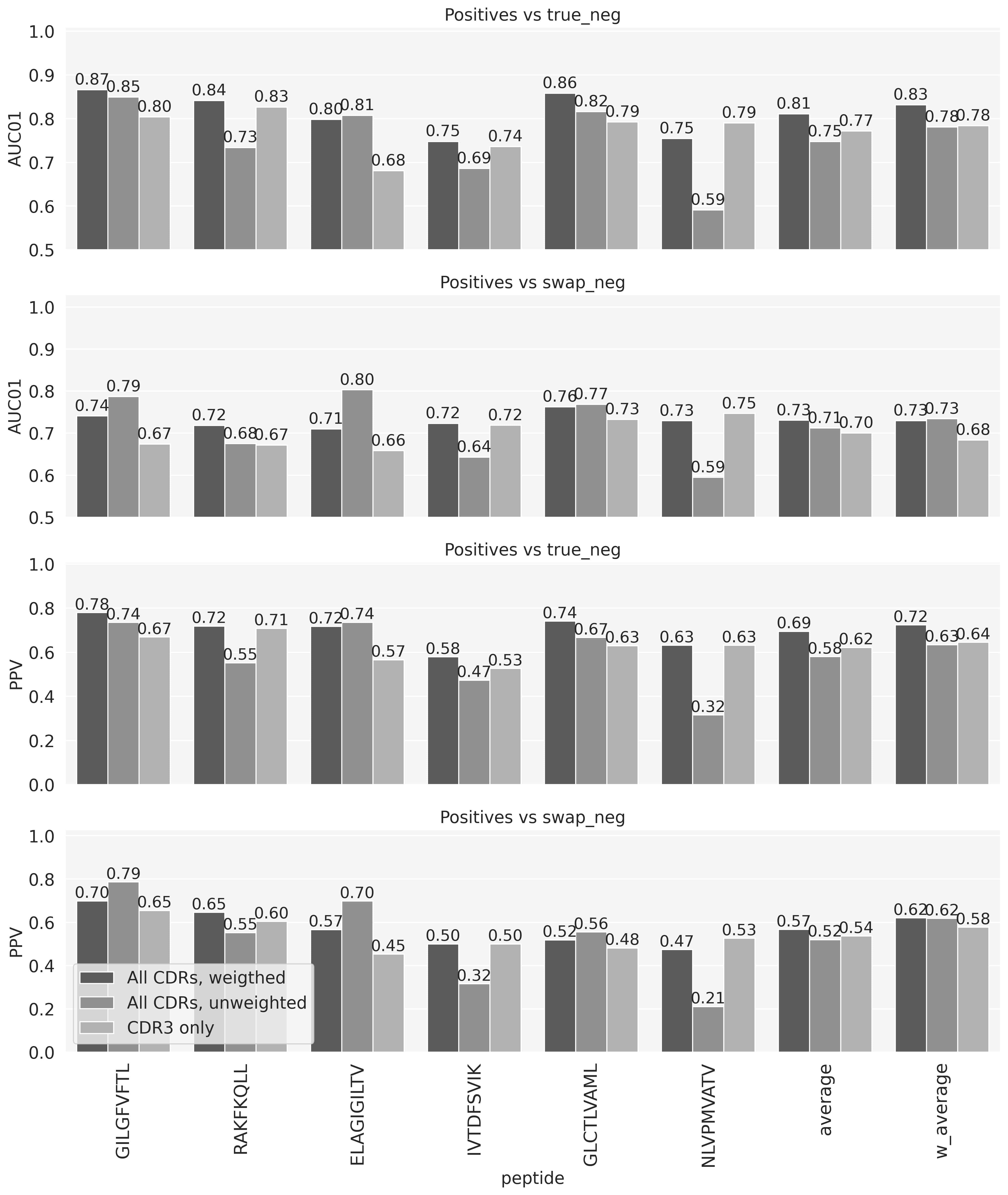

Supplement: Supplementary Figure 1 — Baseline model performance comparison in terms of AUC01 and PPV. The baseline model was used i) with weights [1, 1, 4] on the CDRs; ii) with equal weights on the CDrs; iii) using only CDR3s. The values are given for each peptide, and on the positives vs 10X negatives and positives vs swapped negatives prediction tasks. average and w_average refer to the average and weighted average of the AUC01 (and PPV) across the six peptides. [file Image_1.jpeg]

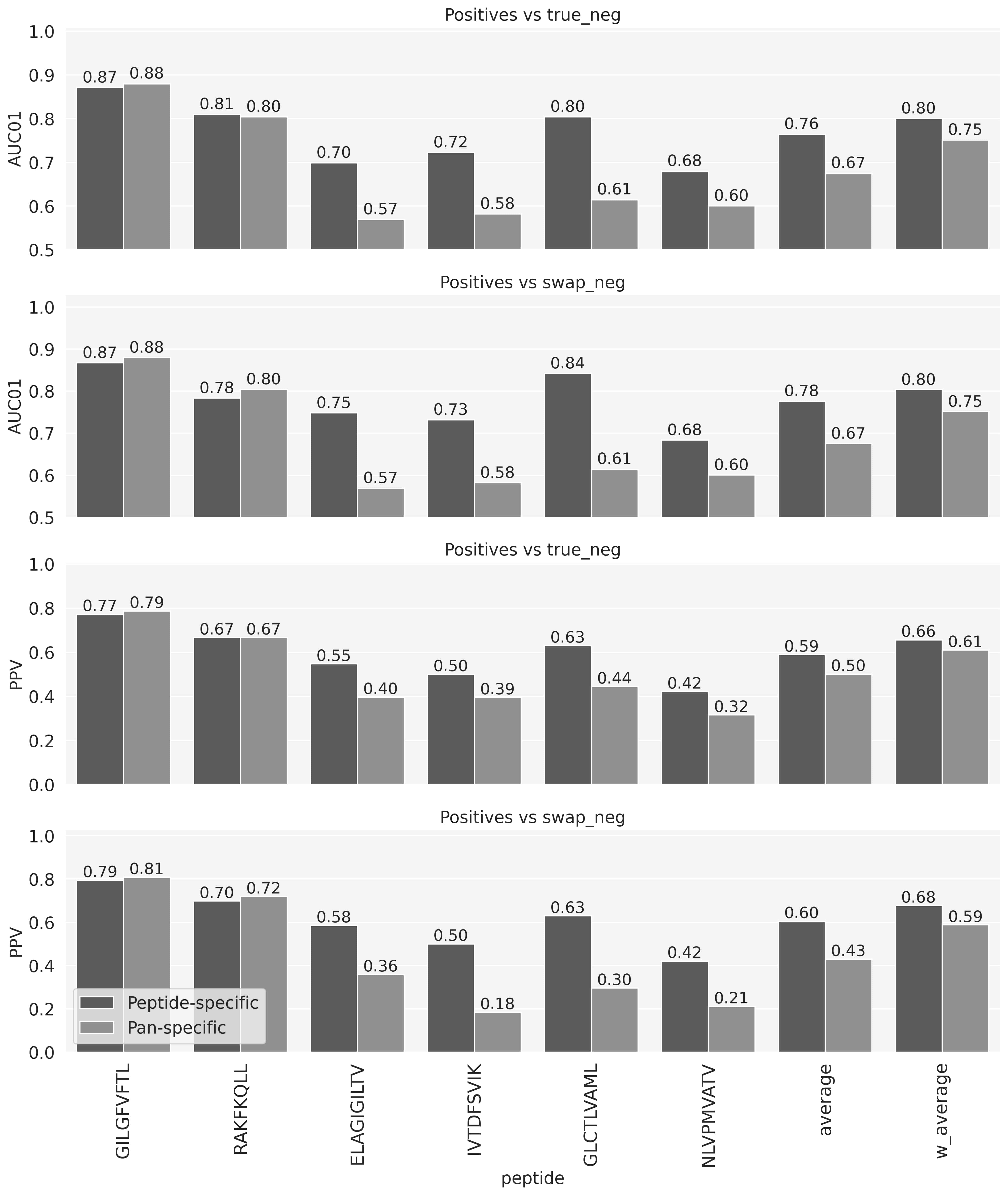

Supplement: Supplementary Figure 2 — AUC01 and PPV values comparison of the NetTCR model trained in a peptide-specific or a pan-specific manner. Performance reported for each peptide, and for positives vs. 10X negatives and positives vs swapped negatives task. Average and weighted average (weighted by the number of positive TCRs for each peptide) performances are also reported. [file Image_2.jpeg]

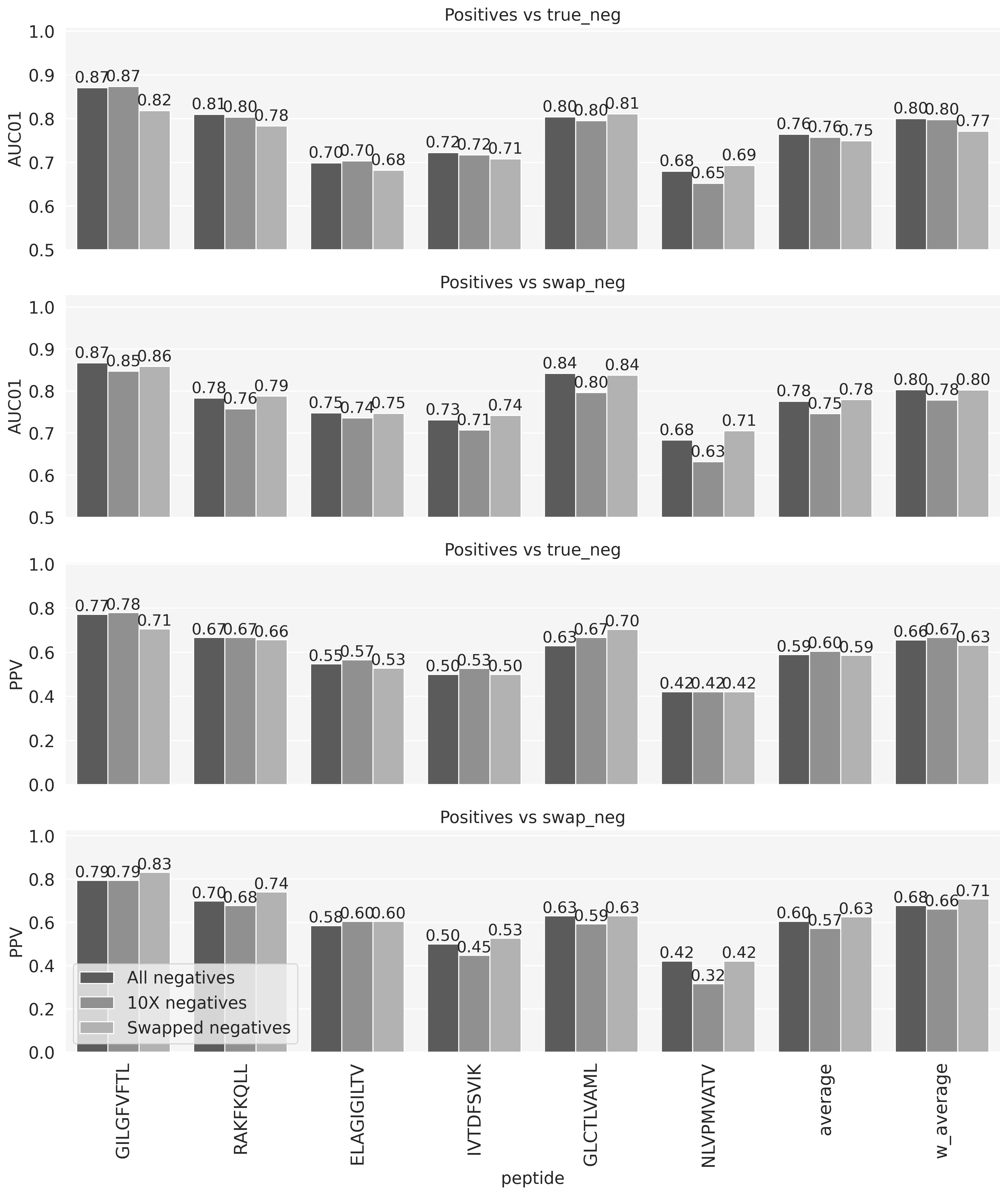

Supplement: Supplementary Figure 3 — Analysis of the different sources of negatives. AUC01 and PPV values for the NetTCR-CDR3 model trained on i) the full dataset, including positives, 10x negative and swapped negatives; ii) positives and 10x negatives only, iii) positives and swapped negatives only. AUC01 and PPV are reported in a peptide-specific manner; the values are also differentiated based on positives versus 10X/swapped negatives predictions. “average” refers to the mean values of AUC01 (and PPV) from each peptide; “w_average” is a weighted average (weighted by the number of positive TCRs) of the values. [file Image_3.jpeg]

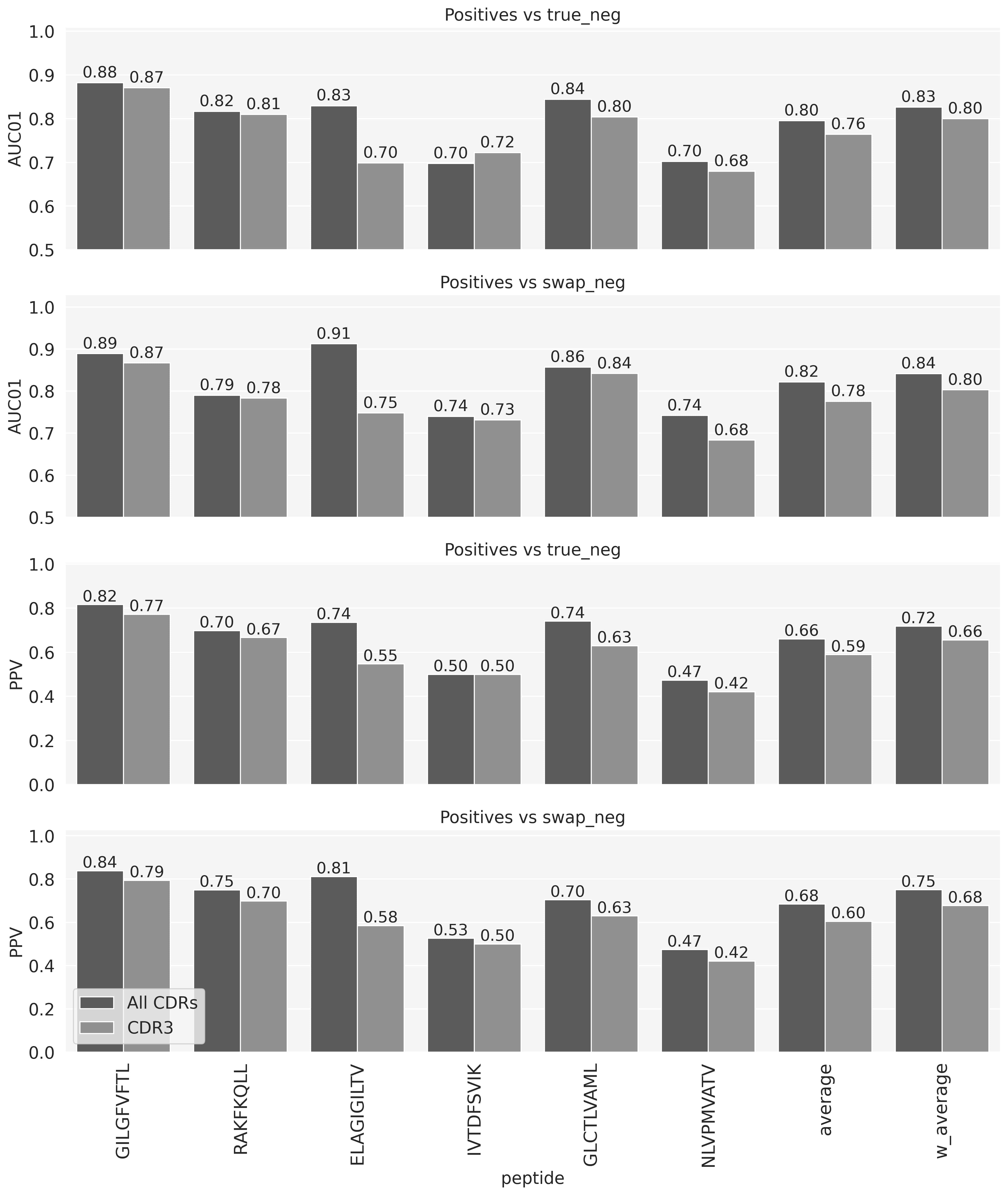

Supplement: Supplementary Figure 4 — Peptide-specific AUC01 and PPV values comparison of the NetTCR models trained using the set of all CDRs or CDR3 only. The predictive power is evaluated for each peptide (average and w_average refer to an average and weighted average, respectively, of the peptide-specific scores). The performance is also differentiated based on the positives vs. 10X/swap negatives predictions. [file Image_4.jpeg]

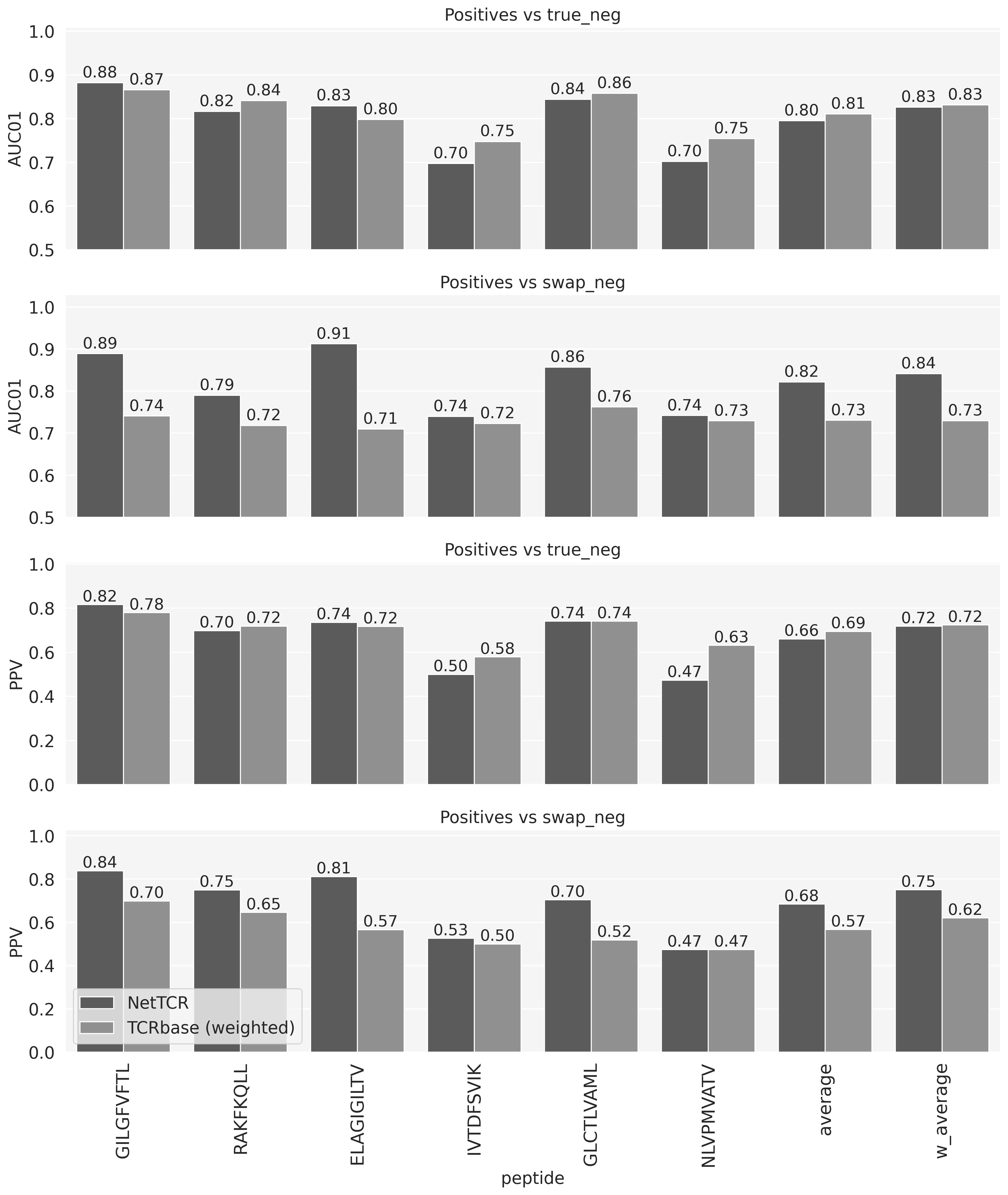

Supplement: Supplementary Figure 5 — NetTCR versus TCRbase. Performance comparison in terms of AUC01 and PPV. The values are reported for each peptide, and differentiated according to the two prediction tasks, positives vs 10x negatives and positives vs swapped negatives. “average” is calculated as an average of the AUC01 (and PPV) of the peptide-specific scores; “w_average” is a weighted average (weighted by the number of positive TCRs) of the peptide-specific scores. [file Image_5.jpeg]
